# Validation of Multi-State EHR-Based Network for Disease Surveillance (MENDS) Data and Implications for Improving Data Quality and Representativeness

**DOI:** 10.5888/pcd21.230409

**Published:** 2024-06-13

**Authors:** Katherine H. Hohman, Michael Klompas, Bob Zambarano, Hilary K. Wall, Sandra L. Jackson, Emily M. Kraus

**Affiliations:** 1 National Association of Chronic Disease Directors, Decatur, Georgia; 2 Department of Population Medicine, Harvard Medical School and Harvard Pilgrim Health Care Institute, Boston, Massachusetts; 3 Commonwealth Informatics, Waltham, Massachusetts; 4 Division for Heart Disease and Stroke Prevention, National Center for Chronic Disease Prevention and Health Promotion, Centers for Disease Control and Prevention, Atlanta, Georgia; 5 Independent Consultant, Public Health Informatics Institute, Task Force for Global Health, Decatur, Georgia

## Abstract

**Introduction:**

Surveillance modernization efforts emphasize the potential use of electronic health record (EHR) data to inform public health surveillance and prevention. However, EHR data streams vary widely in their completeness, accuracy, and representativeness.

**Methods:**

We developed a validation process for the Multi-State EHR-Based Network for Disease Surveillance (MENDS) pilot project to identify and resolve data quality issues that could affect chronic disease prevalence estimates. We examined MENDS validation processes from December 2020 through August 2023 across 5 data-contributing organizations and outlined steps to resolve data quality issues.

**Results:**

We identified gaps in the EHR databases of data contributors and in the processes to extract, map, integrate, and analyze their EHR data. Examples of source-data problems included missing data on race and ethnicity and zip codes. Examples of data processing problems included duplicate or missing patient records, lower-than-expected volumes of data, use of multiple fields for a single data type, and implausible values.

**Conclusion:**

Validation protocols identified critical errors in both EHR source data and in the processes used to transform these data for analysis. Our experience highlights the value and importance of data validation to improve data quality and the accuracy of surveillance estimates that use EHR data. The validation process and lessons learned can be applied broadly to other EHR-based surveillance efforts.

**Summary**

**What is already known on this topic?**

Electronic health record (EHR) data are prone to data quality problems such as missing data. Data quality concerns are often cited as a barrier to using these data for public health surveillance.

**What is added by this report?**

This study offers a framework to inform data quality processes for potential users of EHR data for surveillance. We identified the data quality problems associated with implementing EHR-based chronic disease surveillance and developed remediation strategies.

**What are the implications for public health practice?**

The validation strategies applied in the analysis can be used to evaluate and optimize the integrity of EHR data for public health surveillance.

## Introduction

The Multi-State EHR-Based Network for Disease Surveillance (MENDS) is a pilot project coordinated by the National Association of Chronic Disease Directors and funded by the Centers for Disease Control and Prevention (CDC). It uses clinical data from electronic health record (EHR) systems for public health surveillance by state and local public health departments (1–3). MENDS is built on the Electronic Medical Record Support for Public Health (ESP) surveillance platform, an open-source software designed to transform raw EHR data into usable public health information (eg, incidence of hypertension treatment and control in a specified jurisdiction stratified by age, sex, race, and ethnicity) (4). MENDS transformation tasks include 1) identifying data elements available in the EHR that are relevant to surveillance of a particular health condition, 2) extracting these data, 3) reformatting and mapping the data into a MENDS-specific database, 4) running algorithms to define cohorts and cases of interest, 5) compiling relevant clinical events for each condition, and 6) calculating and visualizing surveillance indicators.

Data quality issues may be present in the raw EHR data or occur at any point in the transformation process (5). MENDS implemented a 5-stage validation process to address the quality of source data and the integrity of the transformation process. The objective of this study was to describe MENDS validation processes across 5 data-contributing organizations and outline steps to resolve data quality issues.

## Methods

### Administration and documentation

The MENDS Coordinating Center administered the validation process according to the MENDS rules of governance (6). Our approach to validation was iterative and interactive. The validation approach was initially informed by previous work done by MDPHnet, a precursor to MENDS, and guidance from CDC and underwent progressive refinements guided by learnings (7). We prioritized 5 chronic disease indicators in ESP for validation: hypertension, smoking, cholesterol, diabetes, and obesity (8). These indicators were chosen because of their importance to CDC and the availability of existing ESP algorithms for immediate implementation. Results from validation activities and observations were shared with data contributors and then discussed to learn more about specific issues and to reach consensus about how to resolve discovered issues. We documented the validation processes and findings overall and for each data contributor, including when each step was started and completed, issues identified, and steps taken to resolve issues before proceeding to the next stage.

Five data-contributing organizations participated in this study, each taking part in the validation process at some point from December 2020 through August 2023. The 5 MENDS data contributors were a health information exchange, a network of community health centers, a nonprofit research organization, a clinical research network, and an enterprise health data warehouse provider — all intermediaries that collate the EHR data of others rather than serve as direct clinical providers themselves. Data contributors to MENDS aggregate data from numerous health systems and clinics, each with differences, strengths, and gaps. These differences arise in part from differences in the core purposes and operations of the data contributors. All data contributors had prior experience with data sharing activities and ETL (extract, transform, and load) processes and validation; however, the MENDS focus on provisioning data for public health chronic disease surveillance was a novel endeavor for them. Regardless of when data contributors joined MENDS, they provided historical data starting from January 1, 2018, up to the present date. Further details on the development of the MENDS pilot, its technical infrastructure, the data sources of contributors, and the network’s governance are published elsewhere (2,9).

### Validation stages

The first 4 stages of the MENDS multistage validation process (Table 1) consist of the internal validation processes to ensure accurate surveillance estimates.

**Table 1:** Description of the Stages of the Validation Process for Multi-State EHR-Based Network for Disease Surveillance (MENDS) Data

Stage of validation	Description
Stage 0: Data confirmation^a^^,^ b	Team gives data contributor a catalog of required data domains to confirm and describe. The MENDS Coordinating Center reviews the results, and any anomalies are discussed with the data contributor so that missing data are identified before an extract, transform, and load (ETL) script is built. This stage was added after multiple partner sites completed initial data loads with missing critical data. Stage 0 was completed by only 1 data contributor.
Stage 1: Extract, transform, and load (ETL) script^b^	SQL (structured query language) scripts are run on the MENDS database to summarize the total counts of patients, encounters, vital signs, laboratories, and prescriptions over time. The MENDS Coordinating Center reviews the results. When issues are identified, the source data and ETL are reexamined, fixes are made as appropriate and feasible to source data or ETL, and data are reextracted and loaded into the MENDS database.
Stage 2: Data characterization	SQL scripts are run on the MENDS database to characterize the distribution of critical data, including calculating minimum, maximum, mean, and median values. The MENDS Coordinating Center reviews the results. Characterization ensures that the MENDS database is providing valid indicators of clinically meaningful population parameters for epidemiology.
Stage 3: Algorithm	Randomly selected patient identifiers are provided to a data contributor to confirm, in their source data, that the algorithm code is correctly identifying conditions as specified. The MENDS Coordinating Center reviews the results. If indicators do not pass at 90%, updates are made to the ETL, source data, or MENDS database, and replacement identifiers are provided.
Stage 4: External indicator algorithm^c^	Partner sites compare MENDS surveillance estimates with surveillance information from alternative comparable data sources such as the Behavioral Risk Factor Surveillance System (BRFSS), National Health and Nutrition Examination Survey (NHANES), or claims-based estimates.

a Stage 0 was introduced later in the project as a quality improvement effort to alleviate issues later in the validation pathway. Only 1 partner site joined MENDS after Stage 0 was established.

b Source data validation steps may occur 1 time, while subsequent validation steps are repeated for each surveillance indicator.

c MENDS validation is a 5-stage process. Stages 0 through 3 are internal validation steps to confirm that the data flows and algorithms work effectively before indicator calculation. Stage 4 is an external validation step that compares indicators with estimates from other data sources. This study focused on internal validations steps and findings, but Stage 4 is included in this table for completeness.

**Stage 0: Data confirmation**. In this stage, data contributors queried their source systems manually to confirm the presence of MENDS-required data (eg, prescription orders, vital signs, laboratory tests) before any exchange of data. Potential data contributors who lacked essential data elements that could not be rectified did not move forward with joining MENDS.

**Stage 1: Extract, transform, load (ETL) validation**. In this stage, the MENDS Coordinating Center examined data completeness, including total numbers of patients, demographic variables (age, sex and gender, race and ethnicity), state, 5-digit zip codes, and total volume of encounters, vital signs, laboratories, and prescriptions over time. Results were verified by data contributor personnel through direct comparison against the source system.

**Stage 2: Data characterization**. In this stage, the MENDS Coordinating Center looked closely at the data attributes used to identify priority conditions by calculating minimum, maximum, mean, and median values for comparison with expected values and those used by other data contributors. The volume and monthly trends of specific diagnostic codes, laboratories, and prescriptions (eg, top 10 diagnoses) were also reviewed.

Although stages 1 and 2 differ in scope, the MENDS Coordinating Center recognized that lower-than-expected volumes in either stage suggest that the source data are incomplete (eg, no laboratory results in the source system) or that the source data are not flowing completely into the MENDS database (eg, selected laboratories are missing due to failure to retrieve all laboratory results from the source system). The MENDS Coordinating Center assessed lower-than-expected values by comparing record counts across data contributors and other implementations of the ESP outside of MENDS, adjusting for total patient population size.

**Stage 3: Algorithm validation**. This stage entailed data contributors reviewing randomly selected patients flagged with each condition (eg, hypertension, smoking status) and condition state (eg, hypertension control, current smoker). MENDS used a limited data set that included a patient identifier. This identifier was passed back to the data contributor, where it was mapped to the fully identifiable patient record, allowing a clinical data specialist at the site to conduct reviews. The primary purpose of stage 3 was to confirm that condition-specific algorithms had been correctly applied, but it also acted as a second check on source data validity. The number of reviews varied by condition, with the intention of balancing the workload of medical record review with the minimum capacity needed to identify data quality errors (Table 2). For each reviewed patient, the data contributor compared the patient’s MENDS classification (eg, hypertension) with the pertinent data elements in the source data (eg, serial blood pressure measurements, diagnosis codes for hypertension, prescriptions for antihypertensive medications) to confirm agreement between MENDS data and the source data.

**Table 2:** Counts of Planned and Completed Stage 3 Validation Reviews by Condition and Outcome in Multi-State EHR-Based Network for Disease Surveillance (MENDS)

Condition	Outcome	Planned	Completed
**Hypertension**	**All**	**100**	**100**
	Clinical hypertension	20	20
	Diagnosed hypertension, controlled	20	20
	Diagnosed hypertension, uncontrolled	20	20
	Diagnosed hypertension, unknown control	20	20
	No hypertension	20	20
**Smoking**	**All**	**80**	**20**
	Never smoker	20	5
	Current smoker	20	5
	Former smoker	20	5
	Status unknown	20	5
**Cholesterol**	**All**	**84**	**20**
	HDL, elevated	7	2
	HDL, healthy	7	2
	HDL, no results in the last year	7	1
	LDL, elevated	7	2
	LDL, healthy	7	2
	LDL, no results in the last year	7	1
	Total cholesterol, elevated	7	2
	Total cholesterol, healthy	7	2
	Total cholesterol, no results in the last year	7	1
	Triglycerides, elevated	7	2
	Triglycerides, healthy	7	2
	Triglycerides, no results in the last year	7	1
**Diabetes**	**All**	**67**	**20**
	Prediabetes	5	0
	Type 1 diabetes, controlled	7	4
	Type 1 diabetes, poor control status	7	3
	Type 1 diabetes, unknown control status	7	3
	Type 2 diabetes, controlled	7	4
	Type 2 diabetes, poor control status	7	3
	Type 2 diabetes, unknown control status	7	3
	Gestational diabetes	20	0
**Obesity**	**All**	**41**	**20**
	Obesity	7	4
	Overweight	7	3
	Healthy weight	7	3
	Height and weight for children	20	10
**Total**	**All**	**372**	**180**

Abbreviations: HLD, high-density lipoprotein; LDL, low-density lipoprotein.

Finally, MENDS recommended that each data contributor conduct stage 4, external indicator algorithm validation, which compares indicators against external data sources, such as state or national chronic disease surveys. We did not address stage 4 validation in this study.

## Results

Among the 5 data contributors participating in MENDS validation during the study period, the median (range) time to complete the internal validation stages 0 through 3 was 15.0 (1.6–20.0) months, with stage 3 taking the most time (median, 9.7 months) (Figure).

**Figure:**
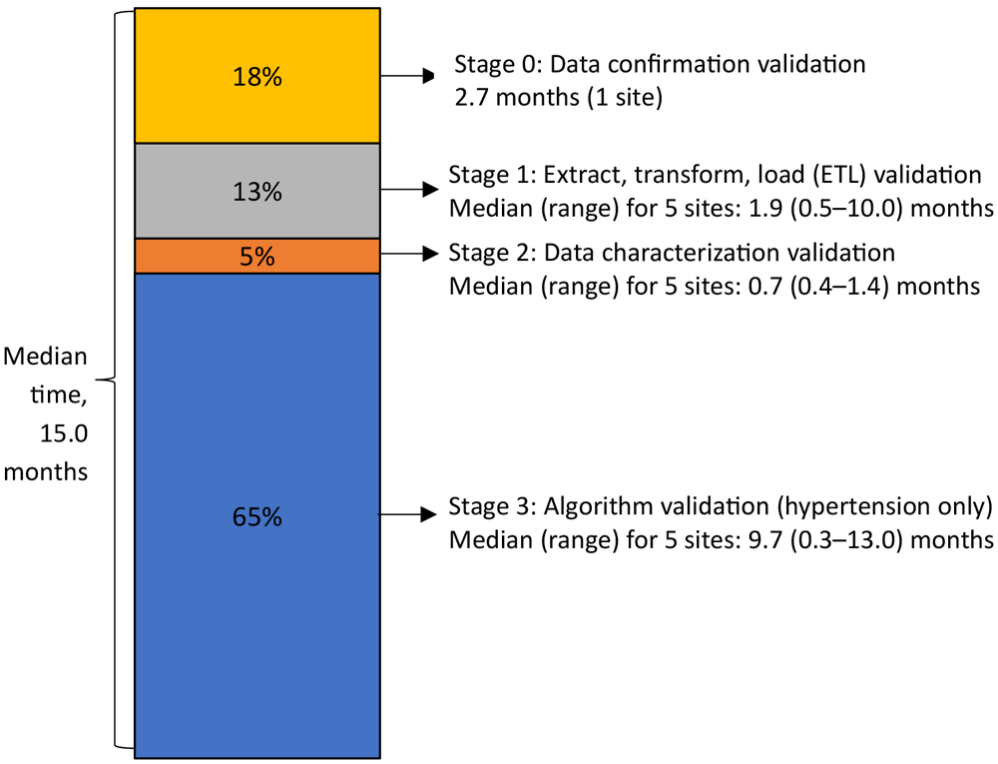
Percentage of total time needed to complete each stage (stages 0–3) of the MENDS internal validation process. Abbreviation: MENDS, Multi-State EHR-Based Network for Disease Surveillance.

The length of time to complete each stage depended on whether issues were identified that data contributors needed to address. For example, if stage 1 was completed with no issues, Stage 2 could proceed immediately. For some issues detected in stage 1, data contributors had to modify their extraction procedure and re-extract data, which could take weeks or months.

Generally, stage 3 reviews took the most time to complete, especially if validation failed because of extraction errors. This led to modifying data extraction processes, re-extracting and reprocessing data, and repeating reviews to confirm resolution. The initial average percentage of hypertension reviews that failed in stage 3 for hypertension was 14% of patients sampled (ranging from 6% to 31% across sites); this rate shrank to 2% (ranging from 0% to 4% across sites) after validation.

### General data quality issues

The most common validation issue was missing data, including lower-than-expected data volume, missing demographic or geographic information, and missing data on patients (Table 3). Several other idiosyncratic data quality issues included incomplete or incorrect mapping of source data values to value sets specified for the ESP data model, errors in source-data processing, incorrect zero values, and data on vital signs that did not connect with an encounter (referred to as “orphan” observations in MENDS).

**Table 3:** Description and Resolution of Representative Data Quality Issues Identified During Validation of Multi-State EHR-Based Network for Disease Surveillance (MENDS)

Data quality issue	Description	Process modified	Resolution
Missing data, low volume	Source data had incomplete historical laboratory data	Source data corrected	Data contributor updated source data and reloaded data in MENDS, which increased laboratory result data volume from 4 million to 69 million events.
	No specific errors were identified	No actions needed	No actions taken.
	Change in pharmacy vendor resulted in storage of prescription orders in a second location in the source data	ETL updated	Data contributor modified ETL to pull prescription orders from both locations and refreshed data in MENDS to increase prescription volume.
	Some health systems were not providing vital sign data to the data contributor	ETL updated to exclude affected systems	Data contributor could not directly resolve this issue; health systems without substantial vital sign data were excluded from MENDS database.
Missing data, incomplete records	Missing data accurately reflected data received from health systems.	Unresolved	Issue was noted, and no further action could be taken to correct this issue in the MENDS database.
	Zip codes were collected by health systems but not consistently shared with the data contributor.	Source data corrected	Data contributor corrected data feeds from its health systems and refreshed data in MENDS, which reduced missingness from nearly half to only 4% of patients.
Missing patients	MENDS patient identifiers did not match a patient in source data, so diagnosis could not be confirmed.	No actions needed	This error results from routine updates of patient identifiers, duplicate removal, and health system or patient offboarding; patient list for review was revised to replace missing patients to complete validation.
Orphan observations	Heights, weights, and blood pressures were submitted without association with an encounter.	ETL updated	To retain these observations for selective use in some algorithms, MENDS database vendor developed a process to create a generic encounter based on available information.
Additional clinical data terminologies	Diagnostic codes were stored using SNOMED instead of ICD-10-CM terminology.	Algorithms updated	MENDS used available terminology mapping tool to convert SNOMED codes to ICD-10-CM when data were loaded into ESP.
Source data processing, mapping	Higher-than-expected proportion of patients were found in low-frequency class of race and ethnicity due to mapping error.	ETL updated	Mapping assignments for race and ethnicity were corrected in the MENDS database.
Incorrect zero values	High frequency of zero values were found in height and weight fields.	Source data corrected	To correct source data, data contributor corrected processing error affecting incoming health system data.
Source data processing, extraction	Weight and height were rounded incorrectly before extraction.	ETL updated	Extraction was updated to correct rounding error.

Abbreviations: ESP, Electronic Medical Record Support for Public Health; ETL, extract, transform, and load; ICD-10-CM, *International Classification of Diseases, 10th Revision, Clinical Modification*; SNOMED, Systematized Nomenclature of Medicine.

**Low data volume.** Two scenarios occurred with low data volume: 1) a site provided consistently lower-than-expected amounts of data compared with similar data feeds (corrected for population size) of other partners or 2) a sudden, dramatic drop in data volume. We found resolvable causes for most low data volume issues. For some data volume fluctuation, we could not identify specific errors. In such cases, data contributors were notified of the anomalies, and both the MENDS Coordinating Center and data contributors continue to monitor for a future clarification of the issue.

**Incomplete records.** We found incomplete data for key variables across all sites to varying degrees. For example, 1 contributor had missing or unknown race for 23% of patients. Discovery confirmed that this rate of missingness accurately reflected the data received from health systems, and thus no further actions were taken. Another contributor found that 5-digit zip codes were missing for roughly half its patients. Investigation confirmed that health systems do consistently collect and store 5-digit zip codes, but that the health system was not always sharing these data. The health system agreed to improve the data feed to the MENDS data contributor, and in subsequent updates, 5-digit zip code missingness decreased to 4%.

**Missing patients**. Multiple data contributors could not find matching patient IDs in their MENDS database or source data during stage 3 validation. Investigation suggested that this issue occurred because of the methods used by MENDS contributors to make quarterly data updates. It is common, during such updates, to delete data previously extracted for MENDS and to generate a new extract containing historical and recent health data. The new data extract also includes newly issued internal identifiers for some or all patients, including those previously in the MENDS database, a deliberate strategy to limit the risk of re-identification. Therefore, patient IDs do not match from 1 cycle to the next, which limits the time available for validation to the period between case identification in ESP and the start of the next refresh cycle.

In addition, as a result of the patient matching and deduplication efforts data contributors perform as part of their data management processes, the loss of 1 or both MENDS patient IDs may occur as records are merged. Merging duplicates improves quality of the source data but may result in mismatched patient IDs after data refresh cycles. Data contributors may also need to remove 1) individual patients, at the request of those patients or 2) all patients from a health system that has ended its data-sharing relationship with the data contributor.

Although untraceable patient IDs can cause validation delays, they are not considered invalidating. To complete a validation review, missing patients can be replaced by using revised validation listings. To alleviate missing patient IDs, validation reviews should ideally be done in a timely fashion between refresh cycles.

### Condition-specific data quality issues

Some validation issues were specific to the chronic disease indicators being validated. A common finding in the MENDS database was the presence of diagnostic codes, heights, weights, and blood pressure values not connected with an encounter, so-called “orphan” diagnostic codes or observations. Orphan observations are unexpected because diagnosis codes and vital signs are typically generated during clinical encounters. Orphan observations can arise from differences in how health systems define encounters in their EHRs or from clinical visits that do not generate an encounter, such as some virtual visits, telephone calls, or nonbillable visits. Stage 3 reviews also found blood pressure, height, and weight observations with nonmatching encounter dates and observation dates, despite having the same encounter ID. Observations and encounters can also become disjointed as the data move through multiple extractions.

The discovery of orphan observations prompted the development of strategies to retain these data for condition determination by creating generic encounters based on available information (“dummy encounters”) and imputing an encounter date from the date of the orphan observation. Although this process allows orphan data to be retained, dummy encounters lack key contextual information such as care setting (eg, inpatient or outpatient). For hypertension, the condition algorithm was modified to exclude orphan observations that occurred on the same patient day as an inpatient encounter.

**Hypertension.** Validation identified some biologically implausible blood pressure values. The hypertension algorithm was updated to exclude systolic blood pressures <30 mm Hg or >300 mm Hg, and diastolic blood pressures <20 mm Hg or >150 mm Hg (10). These changes supported more accurate counts of hypertension overall and improved detection of undiagnosed hypertension and status of hypertension control. In addition, a review of outlier blood pressure values suggested that many came from the inpatient setting. Because hospitalized patients are prone to very high and very low blood pressures owing to clinical circumstances unrelated to chronic hypertension, the hypertension algorithm was updated to exclude inpatient data. Similarly, orphan blood pressure observations collected on the same day as an inpatient encounter were considered to possibly reflect acute illness rather than the chronic state and thus were excluded.

**Smoking.** The volume of smoking data was initially low, raising concerns about missing data. However, it was difficult to estimate missingness because smoking and tobacco use screening practices varied substantially across and within health care organizations. Investigation revealed that smoking data are often stored in multiple locations in the source data. The process to extract smoking data was revised (often multiple times) by each contributor to search for and incorporate smoking data from multiple locations in the source system. This change was particularly relevant for MENDS sites that contributed data via Consolidated Clinical Document Architecture (CCDA) because smoking data are also stored in multiple places within a CCDA. One data contributor that reprocessed CCDAs to look for tobacco information in multiple places identified an additional 900,000 smoking values — changing 100,000 records from never smoker to current smoker and leading to a notable increase in smoking prevalence in the population of this data contributor.

In addition, large volumes of smoking data were stored without a date and, therefore, no time stamp could be attributed to those observations. Smoking data often reside in the EHR in a data domain known as social history, alongside other assessment-related information, and these data can have an unusual structure and relationship with the encounters recorded within the EHR. We opted not to use smoking data without a date, possibly leading to underestimation of smoking prevalence in some jurisdictions.

Smoking status and tobacco use status are 2 distinct but related concepts, and health systems and clinics do not consistently differentiate them. Some screen for one but not the other, and some screen for both and record conflicting information. Stage 3 validation highlighted this difference and required MENDS to add fields for smokeless tobacco products and non–tobacco smoking substances. Some were not consistently using recommended value sets for smoking (11). For example, 1 data contributor had smoking response values of only “current smoker” and “passive smoker,” but not “never smoker” or “former smoker.” We were unable to resolve this source-data issue.

**Idiosyncratic data quality issues.** Errors in source-data processing and mapping were identified throughout the validation process.

For example, stage 1 validation includes generation of a frequency table of the most common diagnostic codes. It revealed that 1 data contributor used Systematized Nomenclature of Medicine (SNOMED) vocabulary (12) to store diagnostic codes. Initially, MENDS algorithms included diagnostic code value sets by using only *International Classification of Diseases, 10th Revision, Clinical Modification* (ICD-10-CM) terminology. To resolve this issue, we created and applied a SNOMED to ICD-10-CM code map (13).

One data contributor’s stage 2 validation showed a higher-than-expected proportion of patients identifying as Alaska Native. This unexpected proportion was due to incorrect mapping of race values from the source data and was resolved by correcting mapping assignments. Another data contributor found tens of thousands of zero values for heights and weights, resulting from errors in the processing of incoming clinical data (before the data were provided to MENDS). The data contributor corrected its inbound processing to resolve this issue.

Stage 3 validation reviews of patients with obesity revealed that 1 data contributor was rounding heights and weights incorrectly. This finding was shared with the data contributor and corrected.

## Discussion

The MENDS data validation process identified multiple data quality issues, including 1) source data issues that could not be resolved, 2) minor issues requiring explanation, and 3) source data issues that were correctable, either during the ETL process to populate the MENDS database or by the MENDS algorithms that identify cases for surveillance. These issues are likely widespread across EHR data repositories and contain generalizable lessons for ensuring the rigor and validity of chronic disease surveillance using EHR data.

MENDS validation is time and resource intensive. Data contributors receive modest funding to participate in MENDS, but the funds are intended to cover multiple aspects of network participation, such as legal agreements, data sharing, technical environment maintenance, and governance involvement, in addition to validation activities (2,9). Validation findings and resolution work also vary by partner sites, complicating project management. We were sensitive to the burden of validation and, when possible, we modified validation processes to address data contributors’ concerns about workload. For example, we decreased the number of stage 3 reviews asked of the sites by about half. Despite this scaling back, the validation efforts still improved data quality, as seen in the reduced error rate (14% to 2%) during stage 3, algorithm validation.

Using a staged approach allowed MENDS and data contributors to track incremental progress. Validation of Stage 0 through 2 identified high-level data quality issues, such as missing data on prescriptions and demographic characteristics, that would affect surveillance estimates for many conditions. Stage 3 reviews of individual cases identified nuanced and often condition-specific data quality problems missed by overall data assessment. In general, issues identified in earlier stages were easier to fix than issues found in later stages. Most issues could be resolved, improving both MENDS surveillance information and potential secondary uses of the source data. Two studies reported similar EHR data quality issues, suggesting that processing costs for EHR data may be higher than anticipated, owing to the need to find and fix discrepancies and inconsistencies (14,15).

Missing data was the most common issue. Most instances could be corrected in the source data or during the ETL process. We implemented stage 0 validation before bringing the last data contributor on board. This stage entailed addressing source data issues before ETL. This contributor had the fewest missing data issues in later validation stages (16).

Stage 1 and 2 validation methods successfully identified obvious missing data issues, but more subtle missing data issues were not always apparent. Determining the presence, cause, and extent of missing information was challenging due to lack of reliable benchmarks. Similarly, in stage 2, the frequency of biologically implausible values was challenging to interpret, because we lacked values for the expected proportion of biologically implausible blood pressures and other measurements in the source data. In Stage 3, we identified more nuanced quality issues specific to each condition. The MENDS validation efforts highlight the importance of an incremental validation approach: start with high-level trends and patterns and narrow to more specific data quality issues.

We found that most data contributors were not routinely performing data quality monitoring for issues that might affect chronic disease surveillance. Data contributors rarely examine their data in the ways required by MENDS validation (eg, total count of encounters, a critical variable for estimating disease prevalence), underscoring the need for independent validation processes before using raw EHR data for public health surveillance.

Some missing data problems could not be resolved because health systems were not consistently collecting the information. For example, missing data on race and ethnicity in clinical data are common (17). Standards on how best to collect and document race and ethnicity data within EHR systems have only recently been established, and many health systems have yet to adopt them (18,19). However, efforts to improve the quality of race and ethnicity data across health systems have resulted in steady improvements. Continuing to support health systems in making these improvements helps ensure accurate information for public health programs and interventions that address health equity. Awareness that the quality of race and ethnicity data can vary widely across systems (20) can also help researchers interpret MENDS surveillance estimates and facilitate use of these data — along with traditional sources of chronic disease surveillance data (21,22).

Information on smoking status is another area that warrants improvement, both in what is collected and where it is recorded in the EHR. The Centers for Medicare & Medicaid Services’ EHR Incentive Program—also known as Meaningful Use program — required the structured capture of data on smoking status, which has since been standardized through the United States Core Data for Interoperability (USCDI) via SNOMED codes (23,24). Despite this standardization, the process of capturing data on smoking status retains some inconsistencies, possibly stemming from the different variables used to record smoking status, which can be subject to interpretation and not mutually exclusive, impeding accurate classification of patients’ smoking status.

How health systems share data with contributors can also affect missingness. Some data contributors receive a large export of data for all patients or all care, while others employ an event notification framework through which only some conditions or types of events are shared. Under the event notification framework, the data contributor sends information from certain patients or from selected encounters. Although patient demographic traits may be available in the EHR, they may not be present in the encounter data sent to the MENDS data contributor (25). A high rate of missing 5-digit zip codes for some contributors was an unexpected data quality issue, because health systems generally collect and update patients’ addresses consistently to facilitate billing. In many EHR systems, a 5-digit zip code is a required field and is auto-populated based on the address. Erroneous zip codes are far more common in EHRs than missing zip codes; large numbers of missing zip codes may indicate a data transmission or mapping issue rather than incompleteness of source data.

### Recommendations

Based on our experience, we plan the following improvements to the validation framework:

1. Expand stage 0 to examine source data more thoroughly before developing the ETL process — to reduce time investigating and resolving errors in later stages.
2. Add a process in stage 1 validation to document how data refreshes occur and their effects on patient IDs to reduce time following up on missing IDs.
3. Expand stage 1 to include details about additional data attributes and routine benchmarking of the expected distribution, range, interquartile range, median, and percentage of missing values pulled either from the literature (when available) or from distributions across MENDS data contributors.
4. Rerun the stage 1 report annually to identify and resolve new data quality issues — introduced by data system updates at the health system or data contributor levels — that affect MENDS data quality.
5. Incorporate care settings and encounter type stratifications throughout validation to better focus issue discovery and resolution efforts.
6. Include state and local public health partners that will use the data in the validation process, when possible, to deepen their understanding of how clinical data are transformed into surveillance information and to build confidence in using EHR-based surveillance information. State and local public health partners could potentially apply the lessons garnered from this process to their engagement efforts with health systems and clinics to enhance the quality of their data, focusing on EHR documentation needs.

### Conclusion

MENDS validation activities were resource intensive but identified multiple data quality issues that could affect the accuracy and representativeness of calculated indicators; most data quality issues could be resolved. Our experience highlights the value and importance of data validation to improve data quality and the accuracy of surveillance estimates using EHR data. The validation process and lessons learned can be applied broadly to other EHR-based surveillance efforts.

## References

[1] Kraus EM , Brand B , Hohman KH , Baker EL . New directions in public health surveillance: using electronic health records to monitor chronic disease. *J Public Health Manag Pract.* 2022;28(2):203–206. 10.1097/PHH.0000000000001501 35100219

[2] Hohman KH , Martinez AK , Klompas M , Kraus EM , Li W , Carton TW , . Leveraging electronic health record data for timely chronic disease surveillance: the Multi-State EHR-Based Network for Disease Surveillance. *J Public Health Manag Pract.* 2023;29(2):162–173. 10.1097/PHH.0000000000001693 36715594 PMC9897452

[3] National Association of Chronic Disease Directors. Multi-State EHR-Based Network for Disease Surveillance (MENDS). Accessed September 2, 2023. https://chronicdisease.org/mendsinfo

[4] ESP/MDPHnet Coordinating Center. Electronic medical record support for public health. Accessed September 2, 2023. https://www.esphealth.org

[5] Gianfrancesco MA , Goldstein ND . A narrative review on the validity of electronic health record-based research in epidemiology. *BMC Med Res Methodol.* 2021;21(1):234. 10.1186/s12874-021-01416-5 34706667 PMC8549408

[6] Public Health Informatics Institute. *Multi-State EHR-Based Network for Disease Surveillance (MENDS) Governance Principles, Policies, and Processes.* Accessed September 2, 2023. https://chronicdisease.org/wp-content/uploads/2023/07/MENDS_Governance_Document_V3.pdf

[7] Vogel J , Brown JS , Land T , Platt R , Klompas M . MDPHnet: secure, distributed sharing of electronic health record data for public health surveillance, evaluation, and planning. *Am J Public Health.* 2014;104(12):2265–2270. 10.2105/AJPH.2014.302103 25322301 PMC4232140

[8] ESP/MDPHnet Coordinating Center. ESP algorithms. Accessed January 18, 2024. https://espnet.atlassian.net/wiki/spaces/EP/pages/93585410/ESP+Algorithms

[9] Kraus EM , Saintus L , Martinez AK , Brand B , Begley E , Merritt RK , . Fostering governance and information partnerships for chronic disease surveillance: the Multi-State EHR-Based Network for Disease Surveillance. *J Public Health Manag Pract.* 2024;30(2):244–254. 10.1097/PHH.0000000000001810 38271106 PMC10811406

[10] Hohman KH , Zambarano B , Klompas M , Wall HK , Kraus EM , Carton TW , . Development of a hypertension electronic phenotype for chronic disease surveillance in electronic health records: key analytic decisions and their effects. *Prev Chronic Dis.* 2023;20:E80. 10.5888/pcd20.230026 37708339 PMC10516201

[11] Centers for Disease Control and Prevention. Public Health Information Network Vocabulary Access and Distribution System (PHIN VADS): current smoking status (NAACCR). Accessed October 25, 2023. https://phinvads.cdc.gov/vads/ViewValueSet.action?id=29668AC8-226F-4E9C-BA9F-23909213248A

[12] SNOMED International. Accessed September 3, 2023. https://www.snomed.org

[13] National Library of Medicine. SNOMED CT to ICD-10-CM map. Accessed September 3, 2023. https://www.nlm.nih.gov/research/umls/mapping_projects/snomedct_to_icd10cm.html

[14] Mohamed Y , Song X , McMahon TM , Sahil S , Zozus M , Wang Z , ; Greater Plains Collaborative. Electronic health record data quality variability across a multistate clinical research network. *J Clin Transl Sci.* 2023;7(1):e130. 10.1017/cts.2023.548 37396818 PMC10308424

[15] Tsiampalis T , Panagiotakos D . Methodological issues of the electronic health records’ use in the context of epidemiological investigations, in light of missing data: a review of the recent literature. *BMC Med Res Methodol.* 2023;23(1):180. 10.1186/s12874-023-02004-5 37559072 PMC10410989

[16] Ruddle RA , Adnan M , Hall M . Using set visualisation to find and explain patterns of missing values: a case study with NHS hospital episode statistics data. *BMJ Open.* 2022;12(11):e064887. 10.1136/bmjopen-2022-064887 36410820 PMC9680176

[17] Proumen R , Connolly H , Debick NA , Hopkins R . Assessing the accuracy of electronic health record gender identity and REaL data at an academic medical center. *BMC Health Serv Res.* 2023;23(1):884. 10.1186/s12913-023-09825-6 37608282 PMC10463428

[18] Flanagin A , Frey T , Christiansen SL ; AMA Manual of Style Committee. Updated guidance on the reporting of race and ethnicity in medical and science journals. *JAMA.* 2021;326(7):621–627. 10.1001/jama.2021.13304 34402850

[19] Flanagin A , Frey T , Christiansen SL , Bauchner H . The reporting of race and ethnicity in medical and science journals: comments invited. *JAMA.* 2021;325(11):1049–1052. 10.1001/jama.2021.2104 33616604

[20] Johnson JA , Moore B , Hwang EK , Hickner A , Yeo H . The accuracy of race & ethnicity data in US based healthcare databases: a systematic review. *Am J Surg.* 2023;226(4):463–470. 10.1016/j.amjsurg.2023.05.011 37230870

[21] Ansari B , Hart-Malloy R , Rosenberg ES , Trigg M , Martin EG . Modeling the potential impact of missing race and ethnicity data in infectious disease surveillance systems on disparity measures: scenario analysis of different imputation strategies. *JMIR Public Health Surveill.* 2022;8(11):e38037. 10.2196/38037 36350701 PMC9685511

[22] Levitt M , Zonta F , Ioannidis JPA . Comparison of pandemic excess mortality in 2020–2021 across different empirical calculations. *Environ Res.* 2022;213:113754. 10.1016/j.envres.2022.113754 35753371 PMC9225924

[23] Centers for Medicare & Medicaid Services. *Eligible Professional Meaningful Use Core Measures Measure 9 of 13.* Last updated May 2014. Accessed February 5, 2024. https://www.cms.gov/regulations-and-guidance/legislation/ehrincentiveprograms/downloads/9_record_smoking_status.pdf

[24] The Office of the National Coordinator for Health Information Technology. US Core Data for Interoperability (USCDI). Health status assessments. Accessed February 5, 2024. https://www.healthit.gov/isa/taxonomy/term/811/uscdi-v4

[25] Yoon J , Mizrahi M , Ghalaty NF , Jarvinen T , Ravi AS , Brune P , . EHR-Safe: generating high-fidelity and privacy-preserving synthetic electronic health records. *NPJ Digit Med.* 2023;6(1):141. 10.1038/s41746-023-00888-7 37567968 PMC10421926

